# Radiomics-guided checkpoint inhibitor immunotherapy for precision medicine in cancer: A review for clinicians

**DOI:** 10.3389/fimmu.2023.1088874

**Published:** 2023-03-01

**Authors:** Huijie Zhou, Qian Luo, Wanchun Wu, Na Li, Chunli Yang, Liqun Zou

**Affiliations:** ^1^ Division of Medical Oncology, Cancer Center and State Key Laboratory of Biotherapy, Sichuan University West China Hospital, Chengdu, China; ^2^ Department of Hematology, the Second Affiliated Hospital Zhejiang University School of Medicine, Zhejiang, China

**Keywords:** radiomics, immunotherapy, precision medicine, pseudoprogression, hyperprogression

## Abstract

Immunotherapy using immune checkpoint inhibitors (ICIs) is a breakthrough in oncology development and has been applied to multiple solid tumors. However, unlike traditional cancer treatment approaches, immune checkpoint inhibitors (ICIs) initiate indirect cytotoxicity by generating inflammation, which causes enlargement of the lesion in some cases. Therefore, rather than declaring progressive disease (PD) immediately, confirmation upon follow-up radiological evaluation after four–eight weeks is suggested according to immune-related Response Evaluation Criteria in Solid Tumors (ir-RECIST). Given the difficulty for clinicians to immediately distinguish pseudoprogression from true disease progression, we need novel tools to assist in this field. Radiomics, an innovative data analysis technique that quantifies tumor characteristics through high-throughput extraction of quantitative features from images, can enable the detection of additional information from early imaging. This review will summarize the recent advances in radiomics concerning immunotherapy. Notably, we will discuss the potential of applying radiomics to differentiate pseudoprogression from PD to avoid condition exacerbation during confirmatory periods. We also review the applications of radiomics in hyperprogression, immune-related biomarkers, efficacy, and immune-related adverse events (irAEs). We found that radiomics has shown promising results in precision cancer immunotherapy with early detection in noninvasive ways.

## 1 Introduction

Immunotherapy using ICIs has been revolutionary in cancer treatment owing to its significant impact on the reactivation of the immune system (1, 2). Unlike traditional cancer treatment approaches, which kill tumor cells directly, ICIs initiate indirect cytotoxicity by generating inflammation and may cause enlargement of the lesion in some cases. Hence, there may be different interpretations of medical imaging for patients undergoing immunotherapy (3).

Medical images contain many quantitative biomedical features based on intensity, shape, size or volume, and texture, which can offer information on the tumor microenvironment and phenotype. These features are difficult to identify by human vision alone.

Radiomics is an emerging field that extracts quantitative features from medical images and converts digital medical images into mineable, high-dimensional data with new high-throughput approaches.

Features extracted in radiomics can be divided into two categories: “semantic” and “agnostic” (4). Semantic features include shape, location, vascularity, speculation, necrosis, attachments, and lepidics, commonly used in *imaging* reports. However, radiomics can quantify these features with computer assistance. Agnostic features include histograms (skewness, kurtosis), haralick textures, laws textures, wavelets, Laplacian transforms, Minkowski functionals, and fractal dimensions. These features can provide intratumoral heterogeneity information through quantitative descriptors (4).

The process of radiomics involves the following discrete steps:

1. Image acquisition (i.e., CT, MR, and PET/CT)
2. Volume of interest (VOI) identification and segmentation: identifying tumors and their surroundings as VOIs and delineating the borders of the volume
3. Feature extraction and qualification: extracting and qualifying high-dimensional features from the VOI
4. Modeling: mining extracted features with artificial intelligence to develop classifier models that aid detection, diagnosis, prognosis assessment, and treatment response prediction

This approach could be applied to any aspect of medical imaging analysis, including immunotherapy, thereby providing a novel noninvasive approach to precision cancer treatment.

**Figure 1:**
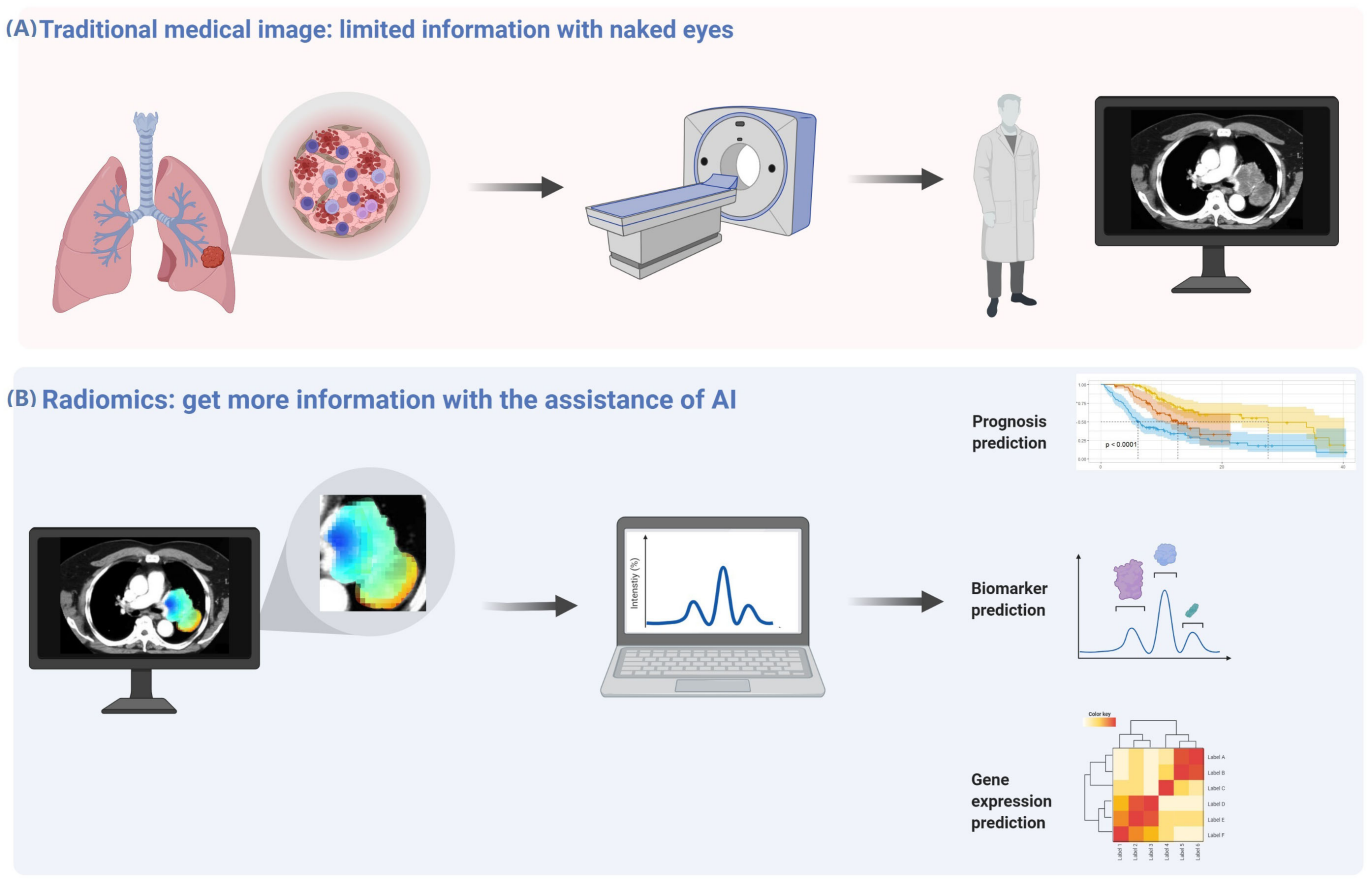
Radiomics analysis can obtain more information from medical images. **(A)** There is genomic and microenvironment heterogeneity within the tumor volume displayed on the imaging, while these fine distinctions cannot be recognizable by the naked eye, even for experienced radiologists. **(B)** These subtle differences can be recognized by radiomics using quantitative assays, allowing microscopic analyses of medical imaging to establish predictive, diagnostic, and prognostic models.

Genomic and microenvironment heterogeneity within the tumor volume is displayed on the imaging, while these fine distinctions cannot be recognizable by the naked eye, even for experienced radiologists. Nevertheless, these subtle differences can be recognized by radiomics using quantitative assays, allowing for microscopic analyses of medical imaging to establish predictive, diagnostic, and prognostic models (**Figure 1**).

This review summarizes radiomics concerning immunotherapy from a clinical perspective. It discusses its potential to predict outcomes, molecular biomarkers, atypical responses, and immune-related adverse events (irAEs) of immunotherapy.

## 2 Prediction of immune-related biomarkers

To date, predictive biomarkers of immune responses are mainly driven by invasive tissue biopsy, while limited biopsy samples may be difficult to provide a holistic picture of the heterogeneity within the tumor and its microenvironment. Radiomics is a powerful auxiliary to conventional invasive biopsies, overcoming the intratumoral heterogeneity within the same patient. The section below describes current progress in immune-related biomarkers using radiomics and how radiomics can overcome these limitations (**Table 1**).

**Table 1:** Summary of Key Studies on the Role of radiomics in predicting the expression of immune-related biomarkers.

Reference	Tumor type	Application	NO. of cases	Imaging modality	Segmention	Segmention software	Machine learning algorithm	Prediction model	Machine learning software	Results	Type of validation
Giulia Mazzaschi (5)	NSCLC	PD-L1	100	CT	Semi-automatically	3d-Slicer	Regression	Multiparametric prognostic model	SPSS, STATA	AUC: 0.91	Independent validation with single-center data
Hectors (6)	HCC	PD-L1	48	MRI	Manual	OsiriX	Logistic regression	Radiomics features	MA TLAB, SPSS	R=0. 41–0.47, p<0. 029	Independent validation with single-center data
Yoon (7)	Advanced lung adenocarcinoma	PD-L1	153	Pretreatment thin section CT	Semi-automatically	Aview Research	Logistic regression	Clinical-Radiomics model	SPSS, MedCalc, R	C-statistic, 0.667	Independent validation with single-center data
JIANG M (8)	NSCLC	PD-L1	399	PET/CT	Semiautomatically	ITK-Snap	Logistic regression and random forest classifier	Radiomics model	Python	The highest AUC, 0.97	Independent validation with single-center data
CHEN (9)	Squamous Cell Carcinoma of the Head and Neck	PD-L1	53	PET/CT	Manual	SPSS	Regression	Textural index of correlation	SPSS	OR, 0.009 (p = 0.015)	Independent validation with single-center data
MU (10)	NSCLC	PD-L1	616	PET/CT	Semiautomatically	ITK-Snap	Deep learning	Deep learning score	MATLAB	AUC, 0.81	Independent validation with multi-center data
Polverari (11)	NSCLC	PD-L1	57	PET/CT	Semiautomatically	LIFEx	Regression	Radiomics features (coarseness and GLZLM_ZLNU)	R	P=0.025,0.035	Independent validation with single-center data
Sun (12)	Advanced solid tumours	Tumour-infiltrating CD8 cells and response to immunotherapy	491	CT	Semiautomatically	LIFEx	Regression	Radiomics signature	R	AUC, 0.67	Independent validation with multi-center data
Tang (13)	NSCLC	PD-L1 and CD3 infiltration	290	CT	Semiautomatically	3D Slicer, IBEX	Hierarchical clustering algorithm	Radiomics model	R	AUC, 0.853	Independent validation with single-center data
LIAO (14)	HCC	Tumor-Infiltrating CD8+T Cells	142	CT	Manual	ITK-SNAP	Elastic net regression	Radiomics model	R	AUC, 0.751	Independent validation with single-center data
CHEN (15)	HCC	Immunoscore	207	MRI	Manual	A.K. software	Regression	Clinical -radiomics nomogram	Python, R	AUC, 0.926	Independent validation with single-center data
Khorrami (16)	NSCLC	TILs	139	CT	Manual	3D SLICER	Linear discriminant analysis (LDA) classifier	Delta-radiomics model	R	AUC=0.88 ± 0.08	Independent validation with multi-center data
HE (17)	NSCLC	TMB	327	CT	Semiautomatically	3D SLICER	Deep learning	Deep learning model	Python, R	AUC= 0.85	Independent validation with single-center data
Veeraraghavan (18)	Endometrial cancers	MMR, TMB	150	CT	Manual	ITK-SNAP	Random forest classifier	Clinical-radiomic model	R	AUC(MMR)=0.78; AUC(TMB)=0.87	Independent validation with single-center data
Golia (19)	Colorectal cancer	MSI	198	CT	Manual	ITK-SNAP	Random forest classifier	Clinical -Radiomics model	MATLAB	AUC=0.80	Independent validation with single-center data
Kather (20)	Gastrointestinal cancer	MSI	81	H&E histology	Manual	Macenko method	Deep learning	Deep learning model	MATLAB, R	AUC=0.84	Independent validation with multi-center data
Wang (21)	Lung adenocarcinoma	TMB	51	CT	Manual	3D-Slicer	Support vector machine (SVM)	Clinical-radiomic model	R	AUC= 0.671	Independent validation with single-center data
Tang (13)	Bladder cancer	TMB	75	CT	Manual	3D-Slicer	Regression	Radiomics model	R	AUC=0.853	Independent validation with single-center data
WEN (22)	NSCLC	TMB; PD-L1	120	CT	Manual	3D Slicer	Regression	Clinicopathological radiomics model	R	AUC(PD-L1) = 0.839 AUC(TMB)=	Independent validation with single-center data
Li (23)	Colorectal cancer	MSS	173	PET/CT	Manual	ITP-SNAP	Regression	Radiomics model	Python	AUC=0.828	Independent validation with single-center data
CAO (24)	Colorectal cancer	MSI	502	CT	Manual	ITP-SNAP	Regression	Clinical Radiomics nomogram	R	AUC=0.898	Independent validation with multi-center data
Pei (25)	Colorectal cancer	MSI	702	CT	Manual	–	Regression	Clinical Radiomics nomogram	R	AUC=0.74	Independent validation with single-center data
Zhang (26)	Rectal cancer	MSI	491	MRI	Manual	ITK-SNAP	Regression	Deep learning model	Python	AUC= 0.868	Independent validation with single-center data
Jiang (27)	Gastric cancer	Immunescore based on immunohistochemistry	1778	CT	Manual	ITK-SNAP	Regression	Radiomics model	R	AUC= 0.766	Independent validation with multi-center data
Xue (28)	Rectal cancer	Immunescore based on immunohistochemistry	133	MR	Manual	ITK-SNAP	Logistic regression	Radiomics model	R	AUC= 0.768	Independent validation with single-center data

### 2.1 Radiomics and programmed cell death ligand 1

As a promising treatment for cancer patients, immunotherapy is not effective for all patients (3, 29, 30). Thus, recognizing the appropriate candidate for immunotherapy is of vital importance. PD-L1 expression examined *via* immunohistochemistry (IHC) is associated with the clinical efficacy of anti-PD-1/PD-L1 therapy. It has been widely applied as a reference to immunotherapy decision-making in most cancer types during clinical practice (29, 31). The expression levels of PD-L1 may change during therapy (32, 33). Due to the existence of intratumoral heterogeneity, the IHC test results of a small number of biopsy samples could not be representative of the whole (34–36).

As the artificial intelligence (AI) field progresses, PD-L1 prediction with radiomics has received increased attention in recent years (**Table 1**). Giulia Mazzaschi et al. established a noninvasive model with computed tomography (CT)-extracted features to predict the level of PD-L1 expression and tumor infiltrating lymphocytes (TILs). They found that texture, effect, and margins were directly associated with PD-L1 expression and TILs. These features also correlated with the prognosis of patients with non-small cell lung cancer (NSCLC) (5). Similarly, researchers have tried to explore the potential of radiomics biomarkers for predicting the immuno-oncologic characteristics of hepatocellular carcinomas (HCC) based on magnetic resonance imaging (MRI). They found that radiomics features, specifically texture feature variance and enhancement ratios, were strongly associated with PD-L1 expression and were predictive diagnostic biomarkers for assessing early HCC recurrence (6).

PET/CT was also applied to predict PD-L1 expression levels. The researchers extracted imaging histology features from PET/CT images of 399 lung cancer patients, of which 24 features were closely related to PD-L1 expression levels. The researchers further developed prediction models based on these features. PET/CT-based prediction model achieves 88% AUC in predicting patients with >50% PD-L1 expression (8).

Radiomics features, in combination with clinical characteristics, may present a better predictive performance. Yoon et al. built a PD-L1 prediction model using both the Rad-score and clinical variables, which turned out to be more accurate than the clinical-variable-only derived model (7).

To date, radiomics remains insufficient to replace IHC testing for the detection of PD-L1. Despite this, the repeatable and noninvasive nature of radiomics analysis may offer additional information for difficult-to-repeat invasive PD-L1 analyses.

### 2.2 Radiomics and TILs

TILs in the tumor microenvironment play an essential part in the immune response against cancer and appear to be associated with the outcome of immunotherapy. Previous studies have indicated that tumor-infiltrating regulatory T cells (Treg) and tumor-associated macrophages (TAMs) induce an immunosuppressive microenvironment that is directly responsible for the failure of immunotherapy, while CD8+ T tumor infiltration is associated with better outcomes to cancer immunotherapy (37).

One of the early attempts to create radiomics signatures aimed at predicting the presence of CD8+ T cells and the clinical efficacy of immunotherapy was conducted by Sun et al. The signature combining eight radiomics features was developed from CT images, genomic data, and ribonucleic acid (RNA) sequencing. The signature was validated on external cohorts to discriminate CD8+ cells and immune phenotypes. Researchers also found that a higher baseline radiomics score is associated with a better response to immunotherapy (12). This study used a radiomics-based biomarker to correlate pathology with prognosis, while the area under the ROC curve (AUC) score for this prediction model was relatively low. In addition, the parameters of image acquisition were not uniform. Therefore, the credibility of the radiomics signature would be affected (12). Another retrospective study revealed that low CT image intensity and high heterogeneity were associated with lower PD-L1 expression and higher CD3 cell infiltration, which was an immune-activated state strongly correlated with favorable overall survival (OS) (13).

Many studies extract radiomics features from pretreatment or posttreatment medical images to predict the TILs associated with the response to immunotherapy (14, 15). “Delta radiomics” can explore changes in the tumor microenvironment before and after immunotherapy. Therefore, it may provide an earlier and more accurate prediction of the efficacy of immunotherapy before visible changes to the naked eye. Khorrami and colleagues explored the potential of radiomics using pretreatment and subsequent CT images from 135 NSCLC patients treated with ICIs. The concordance of the radiomics features with TIL infiltration was confirmed by comparison with TIL infiltration in diagnostic biopsy samples. They reported that delta-radiomics is associated with response and OS in NSCLC patients undergoing ICIs (16).

### 2.3 Radiomics and tumor mutational burden

Previous studies have suggested that TMB is another predictive biomarker for immunotherapy across multiple cancer types, as high TMB is correlated with greater neoantigen and immune infiltration (38).

Researchers have investigated the potential of applying radiomics to predict the TMB status in patients with advanced NSCLC and endometrial and bladder cancers (17, 18, 21, 22) (**Table 1**). He et al. constructed the TMB radiomics biomarker (TMBRB) to predict the pretreatment TMB status. They observed that TMRRB could accurately divide patients into high TMB and low TMB, thus predicting the OS and progression-free survival (PFS) of NSCLC patients treated with ICIs. The predicted treatment efficacy improves when combined with the Eastern Cooperative Oncology Group (ECOG) performance status (17).

Radiomics has the potential value of being a powerful aid in classifying TMB status. Specifically, combining clinical and pathological features may improve prediction performance (14–16). Radiomics may provide sufficient information despite intratumor heterogeneity to assist clinical decisions on immunotherapy.

### 2.4 Radiomics and other predictive biomarkers of immunotherapy

Studies have also explored the prediction of other predictive biomarkers of immunotherapy. Recent studies have evaluated whether radiomics can identify mismatch repair (MMR)/microsatellite instability (MSI) status. These explorations focused on gastrointestinal malignancies based on CT, MRI, and positron emission tomography-computed tomography (PET/CT) images (19, 23–25, 39–43) (**Table 1**).

CAO et al. evaluated whether CT-based radiomics can predict MSI status in 502 patients with colorectal cancer (CRC) based on preoperative contrast-enhanced CT images. They further combined the clinical characteristics with radiomics and then developed a nomogram to predict the MSI status. The predictive performance of the radiomics-clinical nomogram was superior to radiomics only and clinical only (24). Such findings are consistent with another study (25). Combining clinical and pathological features with radiomics may add specificity to the prediction model and contribute to personalized clinical decision-making. The MRI-based MSI prediction model was similarly developed, with an AUC of 0.868 (26). Jiang et al. immunoscored gastric cancer patients based on the immunohistochemical expression of CD3, CD8, CD45 and CD66b and classified the immunoscores using CT-based radiomics model. The radiomics model accurately classified patients with high immunoscore and low immunoscore with an AUC of 0.786 and had the potential to select patients who would benefit from chemotherapy (27).

## 3 Radiomics and response to immunotherapy

The efficacy of immunotherapy has been proven in large-scale, randomized clinical trials and clinical practice (29, 44–51). However, immunotherapy is only partially effective, emphasizing the need for finding noninvasive and accurate predictive biomarkers to target immunotherapy to the appropriate patients (52, 53).

Predictive biomarkers of immunotherapy, including PD-L1 and TMB status, are acquired *via* biopsy, which is invasive, difficult to perform dynamically, and restricted to a small sample of pathological specimens.

Radiomics offers a noninvasive whole-body evaluation of tissue biomarkers. Heterogeneity within the tumor may harbor prognostic information that can be captured and transferred into radiomics features by radiomics analysis. Considerable evidence suggests that radiomics could predict immunotherapy efficacy by recognizing radiomics features associated with response (**Table 2**).

**Table 2:** Summary of Key Studies on the Role of radiomics in predicting the response to immunotherapy.

Reference	Tumor type	ICI	Application	NO. of cases	Imaging modality	Machine learning algorithm		Imaging feature type	Results(response)	Result(survival)	Type of validation
Khorrami (16)	NSCLC	Nivolumab/pembrolizumab/atezolizumab	Predict TILs, response and OS	139	CT		LDA classifier	Delta-radiomics model	AUC=0.88 ± 0.08	HR(OS) :1.64, P = 0.0011, C-Index = 0.72	Independent validation with multi-center data
Trebeschi 2019 (54)	NSCLC, melanoma	Anti-PD1	Predict response and OS	465	CT		Random forest classifier	Radiomics model	AUC=0.76	1-year survival difference: 24%	Independent validation with single-center data
Durot (55)	Metastatic melanoma	Pembrolizumab	Predict OS and PFS	31	CT		Regression	Radiomics features (Skewness at coarse texture scale)	–	HR(PFS)=4.55, p= 0.0089; HR(OS)=6.017, p= 0.016	Independent validation with single-center data
VELCHETI (56)	locally advanced NSCLC	Nivolumab	Predict response	50	CT		SVM classifier	Delta-radiomics (vessel tortuosity metrics)	AUC=0.79	–	Independent validation with single-center data
Mu (10)	NSCLC	ICIs(Mixed)	Predict response	161	PET/CT		Deep learning (small-residual-convolutional-network model)	Deep learning score	AUC=0.81	–	Independent validation with multi-center data
MU (57)	NSCLC	ICIs	Predict durable clinical benefit, PFS and OS	194	PET/CT		LASSO regression	Radiomics nomogram	AUC=0.86	C-indices (PFS)=0.74 C-indices (OS)=0.83	Independent validation with single-center data
BHATIA (58)	Melanoma brain metastases	ICIs	Predict survival	88	MRI		Regression	Radiomics features (Laplacian of Gaussian edge features)	–	HR(OS)=0.68, P= 0.001	Independent validation with single-center data
Ji (59)	Malignant Tumors of the Digestive System	ICIs	Predict response and OS	87	CT		LASSO Regression	Radiomics model	AUC, 0.806	OS: 6.2 vs.13.8 month; p<0.001	Independent validation with single-center data
Khene ZE (60)	Metastatic renal cell carcinoma	Nivolumab	Predict response	48	CT		Logistic regression	Radiomics model	AUC=0.91	–	Independent validation with single-center data
Korpics MC (61)	treatment-refractory adult solid tumors	Multi-site SBRT and Pembrolizumab	Predict response, OS, and PFS	68	CT		Linear elastic-net model (regression)	Radiomics model	OR =10.2, p=0.012	HR(OS)=0.39, P= 0.005; HR(PFS)=0.47, P= 0.013	Phase I clinical trial
Park KJ (62)	Metastatic urothelial carcinoma	ICIs	Predict response	62	CT		Multivariate logistic regression	Radiomics model (heterogeneous distribution and a peripheral enhancing rim pattern)	AUC, 0.87	PFS (p= 0.044); OS (p=0.035)	Independent validation with single-center data
Polverari (11)	NSCLC	ICIs	Predict response	57	18F-FDG PET/CT		Regression	MTV, TLG and radiomics features (volume and heterogeneity)	MTV (p = 0.027); TLG (p = 0.022)	PFS(p=0.002); OS(p=0.049)	Independent validation with single-center data
Valentinuzzi (63)	NSCLC	Pembrolizumab	Predict survival	30	PET/CT		Regression	Radiomics model (Small Run Emphasis is the most dominant feature)	–	AUC= 0.90	Independent validation with single-center data
Wang (64)	Metastatic melanoma	Pembrolizumab or ipilimumab	Predict Response	50	CT		SVM	Delta-radiomics model	AUC= 0.882	–	Independent validation with single-center data
Nardone (65)	NSCLC	Nivolumab	Predict Response	59	CT		Kaplan Meier analysis	Delta-radiomics model	–	OS (26:5months, P=0.002)	Independent validation with multi-center data
Dercle (66)	Melanoma	ICIs	OS	1374	CT		Random forests	Radiomics model	–	AUC= 0.92	Independent validation with multi-center data
Liang (67)	Gastric cancer	PD-1 inhibitors	Predict Response	87	CT		SVM and logistic regression	Radiomics nomogram	AUC= 0.778	–	Independent validation with single-center data
Barabino (68)	NSCLC	ICIs	Predict Response	33	CT		Relative Variation method	Delta-Radiomics	P value <0.05	–	–
Yu (69)	Solid cancers	ICIs	Predict Response	152	Pretreatment CT		LASSO Regression	Radiomics-based nomogram	AUC=0.847	–	Independent validation with multi-center data

Trebeschi et al. developed a model able to predict the response to immunotherapy. They suggested that greater heterogeneous and nonuniform lesions were associated with a better response in NSCLC, possibly with infiltration and inflammation of the tumor, while the sample size of the melanoma cohort was too small to identify optimal imaging biomarkers (54).

Skewness, representing the heterogeneity of a segmented lesion, was a significant independent predictor of OS and PFS, with a higher skewness value linked with poorer survival (55). Similarly, Velcheti analyzed the CT features of 50 NSCLC patients who underwent nivolumab, and they found that vessel tortuosity was an independent predictor of nivolumab’s efficacy (56).

Radiomics features from 18F-FDG PET/CT scans also showed the ability to predict the response to immunotherapy. Several texture features (PET_SRLGE, KLD_SZE) were associated with durable clinical benefits, demonstrating that patients with more heterogeneous tumors might benefit more from immunotherapy. Notably, the prospective cohort validated the model with an AUC of 0.81 (57). However, these results are somewhat inconsistent with the results from prior studies, which indicated that more heterogeneous tumors with CT textures are associated with worse response rates to chemotherapy or radiation.

Other retrospective studies have explored the potential of radiomics in evaluating survival and responses to immunotherapy in different cancer types, including melanoma (58, 66), gastrointestinal malignancies (59, 67), metastatic renal cell carcinoma (mRCC) (60), treatment-refractory adult solid tumors (61), metastatic urothelial carcinoma (62), and NSCLC (11, 65, 68).

The predictive performance of the radiomics model (consisting of small run emphasis and difference entropy) developed by Valentinuzzi et al. was superior to that of PD-L1 and iRECIST, with an AUC of 0.90. Specifically, small run emphasis has the highest predictive performance to discriminate survival, with higher small run emphasis possibly having OS survival from pembrolizumab treatment (63). These results reflected that patients with more homogeneous tumors might benefit from immunotherapy, consistent with a previous study conducted by Polverari et al., where nonresponders exhibited higher tumor heterogeneity at pretreatment CT images (reflected by higher kurtosis and skewness) than responders (11). A study conducted by Mu et al. showed the opposite result. They found that heterogeneous tumors might be more likely to achieve durable clinical responses (57).

These studies have demonstrated that radiomics has the potential to predict the response to immunotherapy and could facilitate clinical decision-making. Despite its great potential, the application of radiomics in clinical immunotherapy is still in its infancy. Reproducibility and standardization are major problems. Studies have already explored the standardized workflow of radiomics (70–74).

## 4 Atypical response

**Figure 2:**
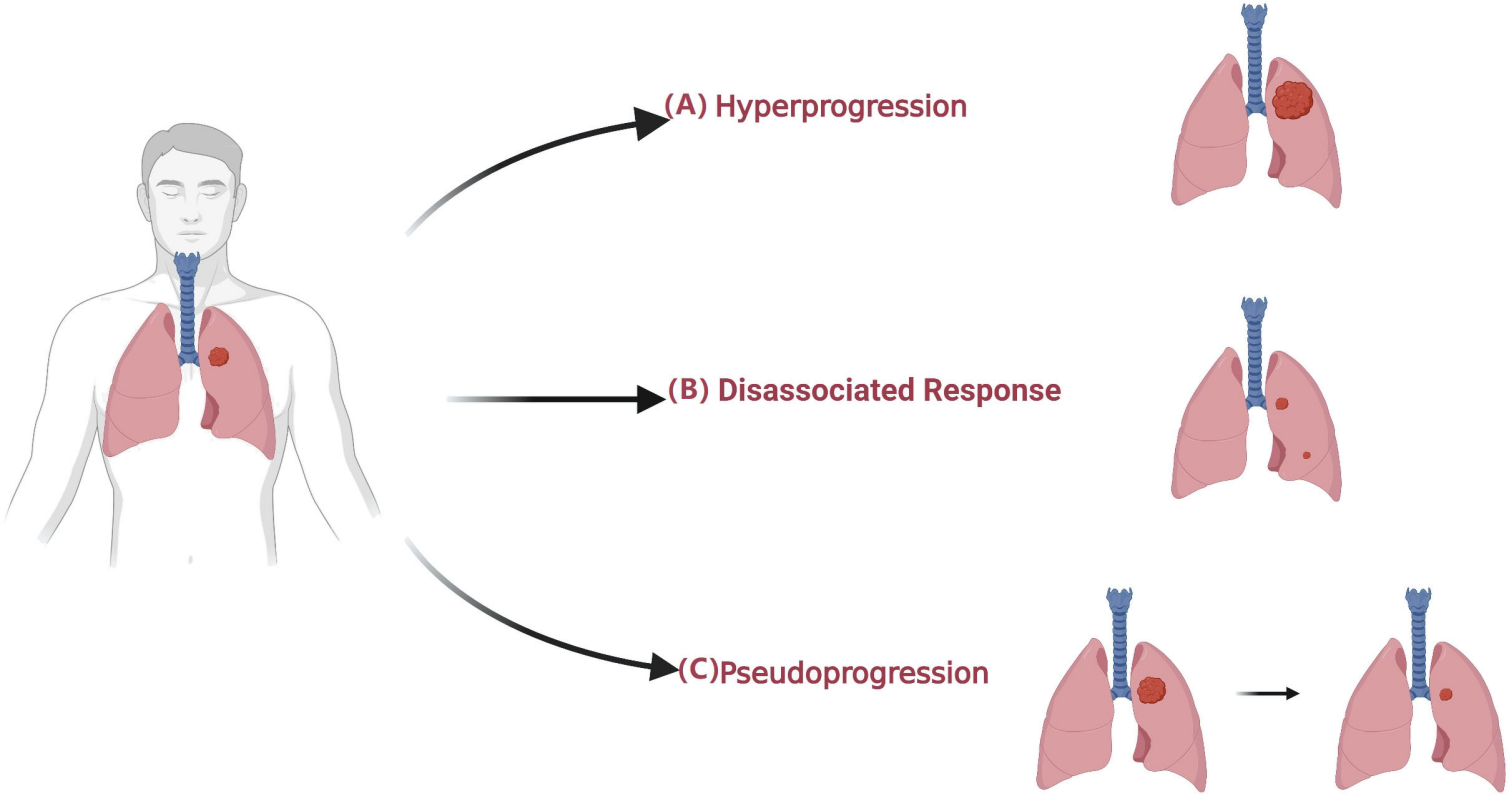
Atypical patterns in patients undergoing immunotherapy. **(A)** Hyperprogression is an atypical response to ICIs with a paradoxical acceleration of tumor growth soon after the initiation of immunotherapy. **(B)** Dissociated Response is defined as a reduction at baseline or increase < 20% in target lesions compared with a nadir in the presence of the new lesion. **(C)** Pseudoprogression is defined as an initial radiographic increase in tumor size or the appearance of new lesions, followed by a response.

In the widespread use of immunotherapy in cancer treatment, unconventional characteristics of response, so-called atypical response, have been observed through imaging (**Figure 2**) (75–77). Atypical patterns of response, including pseudoprogression and hyperprogression, have been demonstrated in clinical trials of ICIs and have prompted the development of immune-related response criteria (78–80). In this part, we summarize how radiomics can support clinical decision-making in light of atypical responses (**Table 3**).

**Table 3:** Summary of Key Studies on the Role of radiomics in predicting atypical responses and irAEs.

Reference	Tumor type	ICI	Application	NO. of cases	NO. of atypical responses/ irAEs	Imaging modality	Machine learning algorithm	Prediction model	Results	Type of validation
pseudoprogression										
JI (59)	Malignant Tumors of the Digestive System	ICIs	Predict Response	87	3	CT	LASSO algorithm	Radiomics model	AUC= 0.76	Independent validation with single-center data
Basler (81)	Metastatic melanoma	ICIs	Differentiate pseudoprogression from true progression	112 patients with 716 metastases	30 lesions	FDG-PET/CT, lesion and routine blood markers	Regression	Blood/radiomics-model	AUC= 0.82	Independent validation with single-center data
He (82)	Solid tumors	ICIs	Discriminating pseudoprogression from true progression	135	34	Pretreatment CT	LASSO regression	Radiomics model	AUC= 0.919	Independent validation with muli-center data
Hyperprogression										
JI (59)	Malignant Tumors of the Digestive System	ICIs	Predict hyperprogression	87	7	CT	LASSO algorithm	Radiomics model (maximum gray has the great significance)	AUC= 0.877	Independent validation with single-center data
MU (10)	NSCLC	ICIs	Predict hyperprogression	616	–	PET/CT	Deep learning	EGFR-DLS	p= 0.037	Independent validation with muli-center data
Vaidya (83)	NSCLC	ICIs	Predict hyperprogression	109	19	CT	Random forest classifier	Peritumoral texture and vasculature patterns	AUC= 0.96	Independent validation with single-center data
Tunali (84)	NSCLC	ICIs	Predicate rapid disease progression	228	15	CT	Logistic regression	Clinical-radiomic model	AUC=0.865	Independent validation with single-center data
He (82)	Solid tumors	ICIs	Discriminating hyperprogression from true progression	135	43	Pretreatment CT	LASSO regression	Radiomics model	AUC=0.933	Independent validation with muli-center data
Gabryś (85)	Metastatic melanoma	ICIs	Predict hyperprogression	56	8	PET/CT	Logistic regression model	Radiomics model	AUC=0.704	Independent validation with single-center data
irAEs										
Colen (86)	Advanced cancers	ICIs	Predicate immune-related pneumonitis	32	2	CT	Unsupervised anomaly detection algorithm	Radiomic model	Accuracy, 100% (p=0.0033)	Independent validation with single-center data

### 4.1 Pseudoprogression

Pseudoprogression is defined as an initial radiographic increase in tumor size or the appearance of new lesions, followed by a response (76, 87, 88). This radiologic effect is due to inflammatory cell infiltration around tumor cells, with an apparent increase in size, and can be confused with tumor cell proliferation (79, 87, 89). To discern pseudoprogression, immune-related response criteria were developed, which suggest further radiological evaluation after four to eight weeks rather than declaring PD immediately (90). A switch to next-line treatment might be delayed while waiting for the confirmatory follow-up evaluation. It is crucial to distinguish pseudoprogression from true disease progression in a timely manner, as it is highly relevant in daily clinical decision-making processes. Therefore, early detection of pseudoprogression is of vital importance.

Texture features extracted from radiological images have been identified to distinguish inflammatory infiltration from tumor cell proliferation. Basler et al. evaluated the capability of PET/CT-based radiomics features, lesion volume, and routine blood markers to differentiate pseudoprogression from true progression at the third month. Of the seven models constructed based on blood, volume, and radiomics, the blood-radiomics model has the best predictive performance, achieving the highest AUC (0.82), and it is a promising biomarker for the early differentiation of pseudoprogression in the third month (81). In a single-center retrospective study, Ji et al. used four radiomics models constructed using contrast-enhanced CT of 87 patients with lung cancer treated with ICIs; of these, model three and model four had AUCs of 0.736 and 0.760, respectively, and were both accurate in predicting responses in two of three pseudoprogression patients (59). Similarly, He et al. used a radiomics approach to identify pseudoprogression from true progression. They extracted intratumoral and peritumoral radiomics features from baseline chest CT scans of 135 patients and built a predictive model. The model had an AUC of 0.96 in the validation set (82). These findings suggest that radiomics can predict pseudoprogression in the course of immunotherapy and may supplement immune-related response evaluation criteria.

### 4.2 Hyperprogression

Hyperprogression is an atypical response to ICIs with paradoxical tumor growth acceleration soon after immunotherapy initiation (91–94). It has been described in numerous cancer types with an incidence of 6%–29% (95, 96). It has been associated with high metastatic burden, significantly shortened survival, and poorer performance status (95, 97–100), thus limiting the potential for administration of other therapies. Identifying high-risk groups is vital, yet there are no predictive biomarkers with apparent effects to identify the risk of hyperprogression (96, 98). As a noninvasive method, radiomics has been explored for the risk stratification of hyperprogression in patients undergoing immunotherapy.

To evaluate the accuracy of a pretreatment CT-based radiomics model in predicting hyperprogression in NSCLC patients treated with ICIs, Vaidya et al. found that peritumoural texture and vasculature patterns in the baseline CT scans positively correlate with hyperprogression. These features had higher expression in hyperprogression than in responders or nonresponders, suggesting that patients with more heterogeneous tumors are more likely to derive durable clinical benefits (83). Consistent with a previous study, a complex model including three radiomics features extracted from the tumor border and several clinical variables was able to predict hyperprogression with an accuracy of 82.28% (84). Other radiomics features, such as the maximum gray value, are intimately linked to the determination of hyperprogression (59). Deep learning models built by Mu et al. have also been reported as possible predictive biomarkers of hyperprogression in NSCLC patients undergoing ICIs. A total of 33.33% of patients with higher EGFR-deep learning scores developed hyperprogression, and deep learning scores were associated with shorter PFS among patients undergoing ICIs (10). Similarly, He et al. developed a prediction model using a CT-based radiomics approach which had an accuracy of 0.933 in identifying hyperprogression from true progression (82). PET/CT-based radiomics has also been attempted for predicting hyperprogression. Gabryś et al. developed a predictive model using PET/CT of patients with metastatic melanoma. CT-based radiological features were shown to be better predictors of hyperprogression than PET-based features (85).

Given the poor prognosis of hyperprogression, it is of great importance for high-risk populations to be screened before initiating immunotherapy. The remarkably accelerated development of radiomics in immunotherapy suggests that radiomics could be used to stratify the risk of hyperprogression. These findings warrant further exploration.

## 5 Radiomics and irAEs

IrAEs associated with immunotherapy, resulting from activating an immune response against healthy tissues, may involve almost every organ and system (101, 102). Timely diagnosis and prompt management depending on its severity, with a proper suspension of ICIs or corticosteroid treatment, is vital. If left untreated, irAEs could develop into life-threatening complications. Therefore, early diagnosis and monitoring of irAEs are crucial for radiologists.

Medical imaging, including CT, ultrasonography, magnetic resonance imaging, X-rays, and PET/CT, was used to detect irAEs. In a retrospective study with a small sample size, Mekki et al. found that 74% (19) of irAE patients (55) showed abnormalities on medical imaging and could be diagnosed by radiologists. The rates of enterocolitis, hypophysitis, thyroiditis, hepatitis, arthralgia or arthritis, lung/mediastinum side effects, and pancreas range from 28% to 100% (103).

The detection of irAEs generally depends on blood test indicators, clinical manifestations, and imaging characteristics. However, radiomics can help in the identification of early invisible signs of irAEs in medical imaging. Colen et al. utilized a radiomics approach to predict the risk for immune-related pneumonitis. They extracted 1860 radiomics features from baseline chest CT scans of 32 patients treated with ICIs, of whom two developed immune-related pneumonitis. Selective radiomics features were utilized to develop the predictive model of the subsequent development of pneumonitis. This model correctly identified the two patients who developed immune-related pneumonitis (86). Despite the noninvasive and impersonal nature of radiomics, studies relevant to the early detection of irAEs are rarely reported (**Table 3**).

## 6 Discussion

With the rapid development and application of artificial intelligence in medicine, radiomics may become a valuable tool in clinical decision-making. Here, we focus on the exciting and innovative space of radiomics to solve the problems in immunotherapy and discuss how radiomics serves as a means to support the precision design of immunotherapy, especially in pseudoprogression and hyperprogression.

Radiomics represents a potential noninvasive and feasible strategy in clinical decision-making that can ensure timely access to results and minimize the bias caused by localized tissue sampling from heterogeneous tumors. In addition, radiomics can potentially be applied to daily clinical practice to monitor responses and side reactions to ICIs.

These advantages provide convenience for clinical diagnosis and treatment, such as noninvasive biopsy, differentiating pseudoprogression from true progression, risk stratification for hyperprogression, immune-related response assessment, and so on. Current studies have indicated that the potential of radiomics in immunotherapy is substantial.

While the results of recent radiomics research are promising, they remain insufficient for its widespread application in daily clinical practice, and radiomics cannot replace biopsy or iRECIST in clinical application at this stage. Limitations and challenges in terms of practical application are not neglectable, and reproducibility presents the most significant challenge.

The heterogeneity of articles exploring radiomics features is an important issue that limits the generalization of the role of radiomics in daily clinical practice, as different imaging modalities are studied (CT, MRI, PET/CT) in different clinical settings (several different neoplasms at various stages of disease) with different “a priori” expected responsivities to immunotherapy. Due to the complexity of radiomics, few studies can be wholly reproduced, thus inhibiting the widespread use of radiomics in clinical practice (104).

Reproducibility remains a huge obstacle in the pace of clinical application Guidelines were established to standardize protocol and analysis of radiomics research (70, 105). The radiomics quality score (RQS) (106) and Individual Prognosis or Diagnosis (TRIPOD) (107) were developed to bridge this gap. Moreover, ongoing single and multicenter prospective randomized clinical trials are needed to improve and validate reliability and reproducibility (**Table 4**). Integration and analysis of radiomics features with genomics, proteomics, and other omics data would provide additional information in precision medicine by uncovering microlevel features (105, 108).

**Table 4:** Clinical trials of radiomics in immunotherapy.

NCT Number	Diseases	Prediction	Enrollment	Study Designs	Start Date	Status
NCT04364776	Lung Cancer	Response to Chemoradiation and Durvalumab	70	Prospective	2020/4/15	Recruiting
NCT04452058	Lung Cancer	Assisting Surgery Decision and Predicting Immunotherapy Response	500	Retrospective	2019/8/1	Recruiting
NCT04792437	Glioma	TMB, OS, PFS	120	Other	2021/3/10	Recruiting
NCT04984148	NSCLC	Predicting the Efficacy of Immunotherapy	70	Prospective	2019/2/1	Recruiting
NCT04994795	NSCLC	Response to Chemotherapy and/or pembrolizumab	4000	Other	2022/8/21	Not yet recruiting
NCT03305380	NSCLC	Identify Patients at Risk for Developing immune-related Pneumonitis	637	Other	2017/9/1	Completed
NCT03712566	Squamous cell cancer of the head & neck, esophagus, or anal canal	Identify genomics and response to immunotherapy	39	Prospective	2018/11/6	Active, not recruiting
NCT04007068	NSCLC	Immunotherapeutics Response and survival	31	Prospective	2017/1/1	Unknown status
NCT04079283	Solid Tumor	Immunotherapeutics Response	285	Retrospective	2017/1/1	Completed
NCT04193956	Melanoma NSCLC	Immunotherapeutics Response	3500	Prospective	2018/8/1	Recruiting
NCT04243720	Solid Tumor	Immunotherapeutics Response	100	Prospective	2020/8/26	Recruiting
NCT04314349	Aerodigestive tract cancers	Identify genomics and pathological features, response to immunotherapy	100	Prospective	2020/8/1	Recruiting

Radiomics provides a window of opportunity for precision medicine of immunotherapy by analyzing microscopic medical imaging in a noninvasive, efficient, economical, and rapid fashion. In the foreseeable future, we envision that radiomics will be widely applied to clinical decision-making and will serve as the impetus for the next major breakthroughs in precision medicine. However, at this stage, there are still significant challenges in the process of clinical translation and application, and further refinements are warranted.

## Author contributions

HZ, QL, WW, NL, CY and LZ: Literature search, concepts development, manuscript drafts. All authors contributed to the article and approved the submitted version. All figures created with BioRender.com.

## Glossary

**Table:**

ICIs	Immune checkpoint inhibitors
PD-L1	Programmed cell death ligand 1
ir-RECIST	Immune-related Response Evaluation Criteria in Solid Tumors
TILs	Tumor infiltrating lymphocytes
AI	Artificial intelligence
NSCLC	Non-small cell lung cancer
HCC	Hepato-cellular carcinomas
MRI	Magnetic resonance imaging
IHC	Immunohistochemistry
RNA	Ribonucleic acid
AUC	Area under ROC curve
OS	Overall survival
Treg	Regulatory T cells
TAMs	Tumor associated macrophages
TMB	Tumor mutational burden
TMBRB	TMB radiomics biomarker
PFS	Progression free survival
ECOG	Eastern Cooperative Oncology Group
MMR	Mismatch repair
MSI	Microsatellite instability
PET/CT	Positron emission tomography computed tomography
CRC	Colorectal cancer
mRCC	Metastatic Renal-Cell Carcinoma
PD	Progressive disease
irAEs	Immune-related adverse events
